# Risk prediction of cardiovascular disease using machine learning classifiers

**DOI:** 10.1515/med-2022-0508

**Published:** 2022-06-17

**Authors:** Madhumita Pal, Smita Parija, Ganapati Panda, Kuldeep Dhama, Ranjan K. Mohapatra

**Affiliations:** Department of Electronics and Communication Engineering, C. V. Raman Global University, Bidyanagar, Mahura, Janla, Bhubaneswar, Odisha 752054, India; Division of Pathology, ICAR-Indian Veterinary Research Institute, Izatnagar, Bareilly 243122, Uttar Pradesh, India; Department of Chemistry, Government College of Engineering, Keonjhar, Odisha 758002, India

**Keywords:** cardiovascular disease, machine learning algorithms, *K*-nearest neighbour, multi-layer perceptron

## Abstract

Cardiovascular disease (CVD) makes our heart and blood vessels dysfunctional and often leads to death or physical paralysis. Therefore, early and automatic detection of CVD can save many human lives. Multiple investigations have been carried out to achieve this objective, but there is still room for improvement in performance and reliability. This study is yet another step in this direction. In this study, two reliable machine learning techniques, multi-layer perceptron (MLP), and *K*-nearest neighbour (K-NN) have been employed for CVD detection using publicly available University of California Irvine repository data. The performances of the models are optimally increased by removing outliers and attributes having null values. Experimental-based results demonstrate that a higher accuracy in detection of 82.47% and an area-under-the-curve value of 86.41% are obtained using the MLP model, unlike the K-NN model. Therefore, the proposed MLP model was recommended for automatic CVD detection. The proposed methodology can also be employed in detecting other diseases. In addition, the performance of the proposed model can be assessed via other standard data sets.

## 1 Introduction

Health is a crucial part of everyone’s life. However, owing to multiple reasons like unhealthy lifestyles, work stress, psychological strain, and external factors such as pollution, hazardous work environment, and lack of proper health services, millions of people worldwide fall prey to chronic ailments like cardiovascular diseases (CVD), which affect both the heart and blood vessels, resulting in death or disability. In recent years, it was reported that the majority of human deaths were due to CVD [1,2]. The associated conditions are hypertension, thromboembolism, hyperlipidaemia, and coronary heart disease, which culminate in heart failure. Hypertension is the primary cause of CVD [3]. In 2012, 7.4 million people were reported to have died from coronary heart disease, while 6.7 million people died from stroke [4]. The World health Organization estimates that nearly 17 million people die every year from CVDs, which accounts for approximately 31% of global deaths. Early diagnosis of CVD can potentially cure patients and save innumerable lives. Diagnosis and treatment of patients at early stages by cardiologists remain a challenge. Every traditional CVD risk-assessment model implicitly assumes each risk factor related to CVD outcome in a linear fashion. Such models have a tendency to oversimplify complex relationships, including several risk factors with non-linear interactions. Multiple risk factors should be properly incorporated, and more correlated nuances between the risk factors and outcomes should be determined. To date, no large-scale study has used routine clinical data and machine learning (ML) in prognostic CVD assessment. The goal of this study is to determine if ML can enhance cardiovascular risk prediction accuracy in population primary care at large and find out which ML algorithm result had fairly high brevity. In recent years, multiple ML-based CVD detection models have been proposed. A review of previous studies is presented to identify the research problem and objective of each study. ML helps a cardiologist to predict diseases at an early stage and treat the patient accordingly. There are many ML techniques such as support vector machines [5], artificial neural networks, decision trees [6], and *K*-Nearest Neighbour (*K*-NN) [7], each with its strengths and weaknesses. These methods have been applied in broader areas like in predicting liver [8,9], human heart (echocardiogram signals) [10,11], and skin diseases [12,13,14]. Results of each technique differ owing to several constraints. Observations from related studies reveal that there is further scope for the development of automated CVD detection using other ML models that provide improved performance. This study contains an in-depth statistical analysis of input data sets to understand the effects of data range on the CVD predictions. It includes a correlation study of categorical and continuous features of patients. In addition, data visualization and scatter plots for pairs of important features were obtained to understand the significance of the correlation between important features. These are discussed and analysed in the results section.

## 2 Materials and methods

The goal of this study was to determine whether or not a patient would develop CVD if a set of clinical information is available. The confusion matrix of each technique was obtained, and out of 303 occurrences in the data set, 243 (80%) were used to train the two models. To test the trained models, 60 instances were fed to know the class. This study intends to predict the likelihood of developing CVD via a computerized prediction route that can be useful to health professionals. The materials required for CVD detection are the test data of patients from publicly available standard CVD data from the UCI repository [15]. The classification algorithms used are MLP and *K*-NN. Generally, the method comprises training of the proposed model via respective learning algorithms using relevant input test data of patients and then validating these models based on test data of patients. Finally, performance measurements are evaluated and compared.

The following steps are carried out to predict CVD:

Step 1: Relevant CVD data set is first collected from the UCI repository.

Step 2: Data samples are pre-processed by eliminating null values, filtering for denoizing, and removing outliers present in samples.

Step 3: Attributes which are more useful in CVD forecasting are selected, and strongly correlated features are dropped.

**Figure 1:**
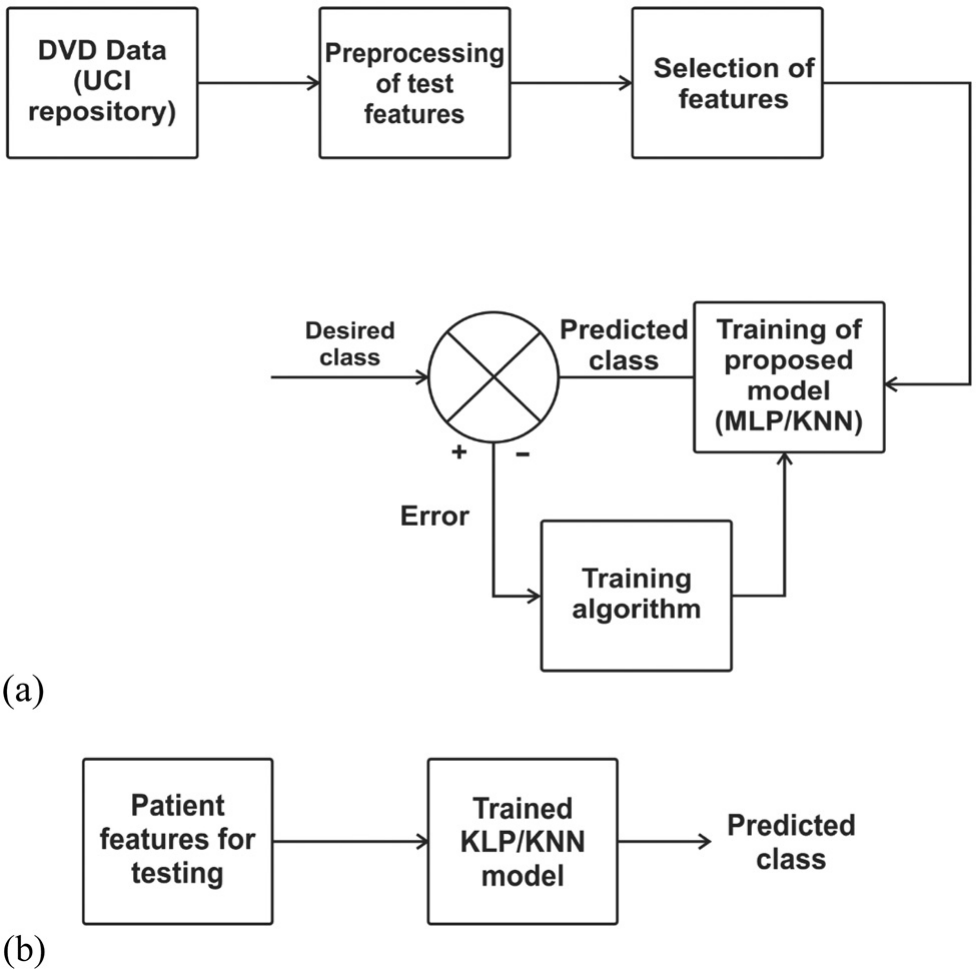
Methodology employed in developing a CVD detection model: (a) training phase of ML models and (b) testing phase of ML models.

Step 4: Two ML algorithms that are simple but effective are chosen to classify the selected features based on Figure 1.

Step 5: Various performance measures are evaluated to compare and find the better method.

### 2.1 Data source

**Figure 2:**
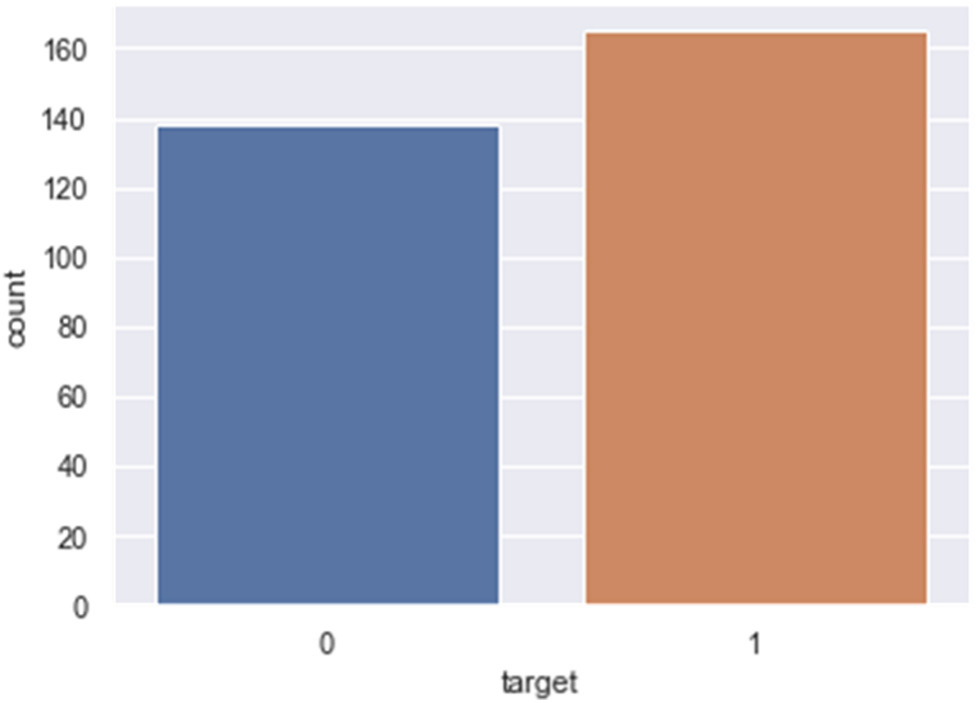
Count plot of patients with CVD (1) and without CVD (0).

The CVD data set used for developing the detection models was taken from the University of California Irvine (UCI) repository [15] and has been converted into a.csv comma-separated file. It contains 303 samples and 76 attributes. Only 13 important test attributes (age, sex, chest pain (cp), resting blood pressure (trestbps), cholesterol (chol), fasting blood sugar (fbs), resting electrocardiographic result (Restecg), maximum heart rate (thalach), exercise-induced angina (exang), ST depression (old peak), slope of peak ST segment (slope), number of major vessels (Ca), thallium stress result (thal)), and one target output (1 = patient having CVD, 0 = patient not having CVD) have been considered out of the 76 attributes to train and test the model. These are presented in Table A1. Our target value taken is whether a person has CVD (near to 1) or does not have CVD (close to 0). The data set was imbalanced as 165 patients had CVD and 138 patients were normal (Figure 2).

**Figure 3:**
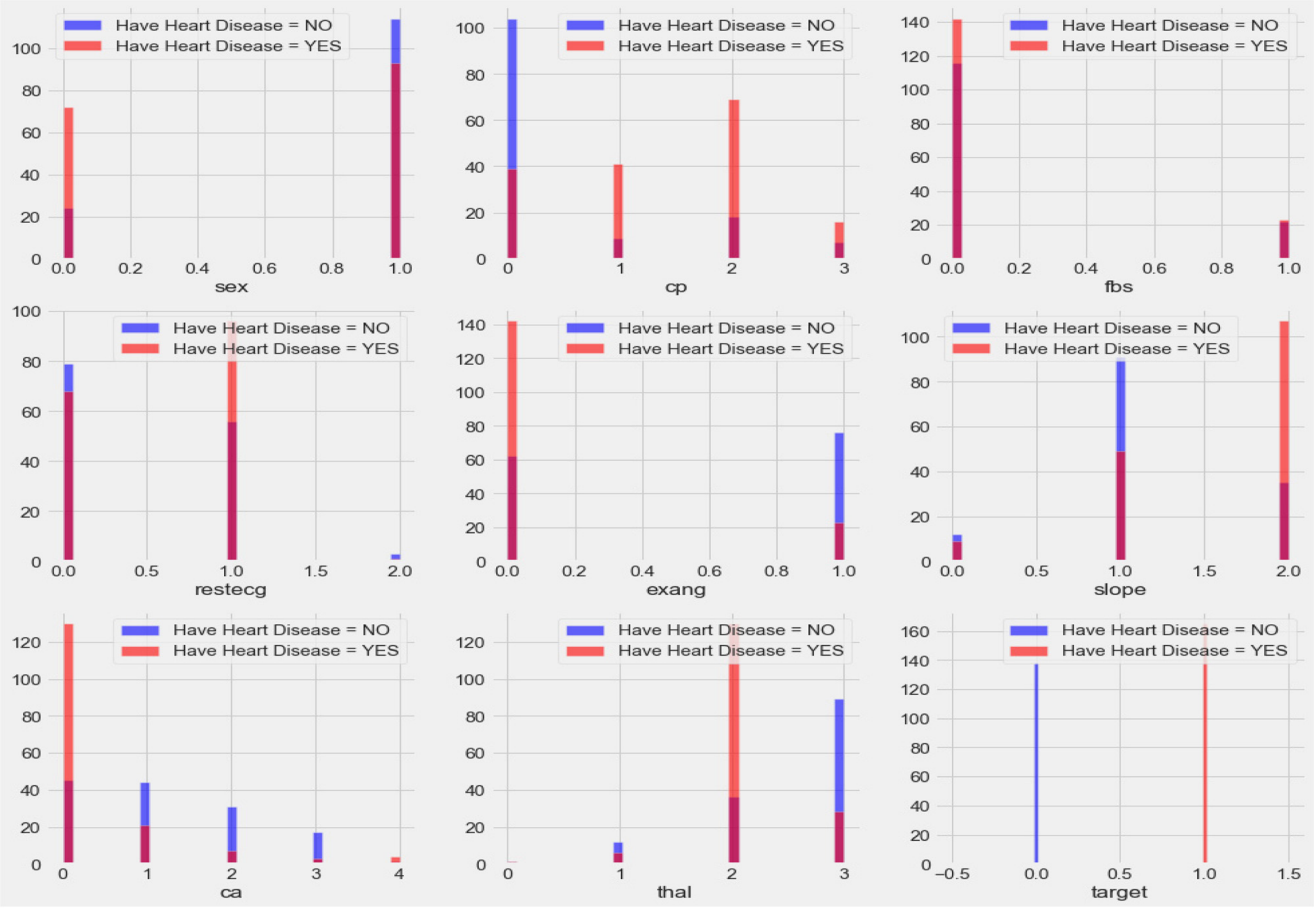
Correlation plot for CVD data set categorical features (cp: chest pain, fbs: fasting blood sugar, Restecg: resting electrocardiographic result, exang: exercise-induced angina, slope: slope of peak ST segment, Ca: number of major vessels, thal: thallium stress result, and target output (1 = patient having CVD, 0 = patient not having CVD)).

**Figure 4:**
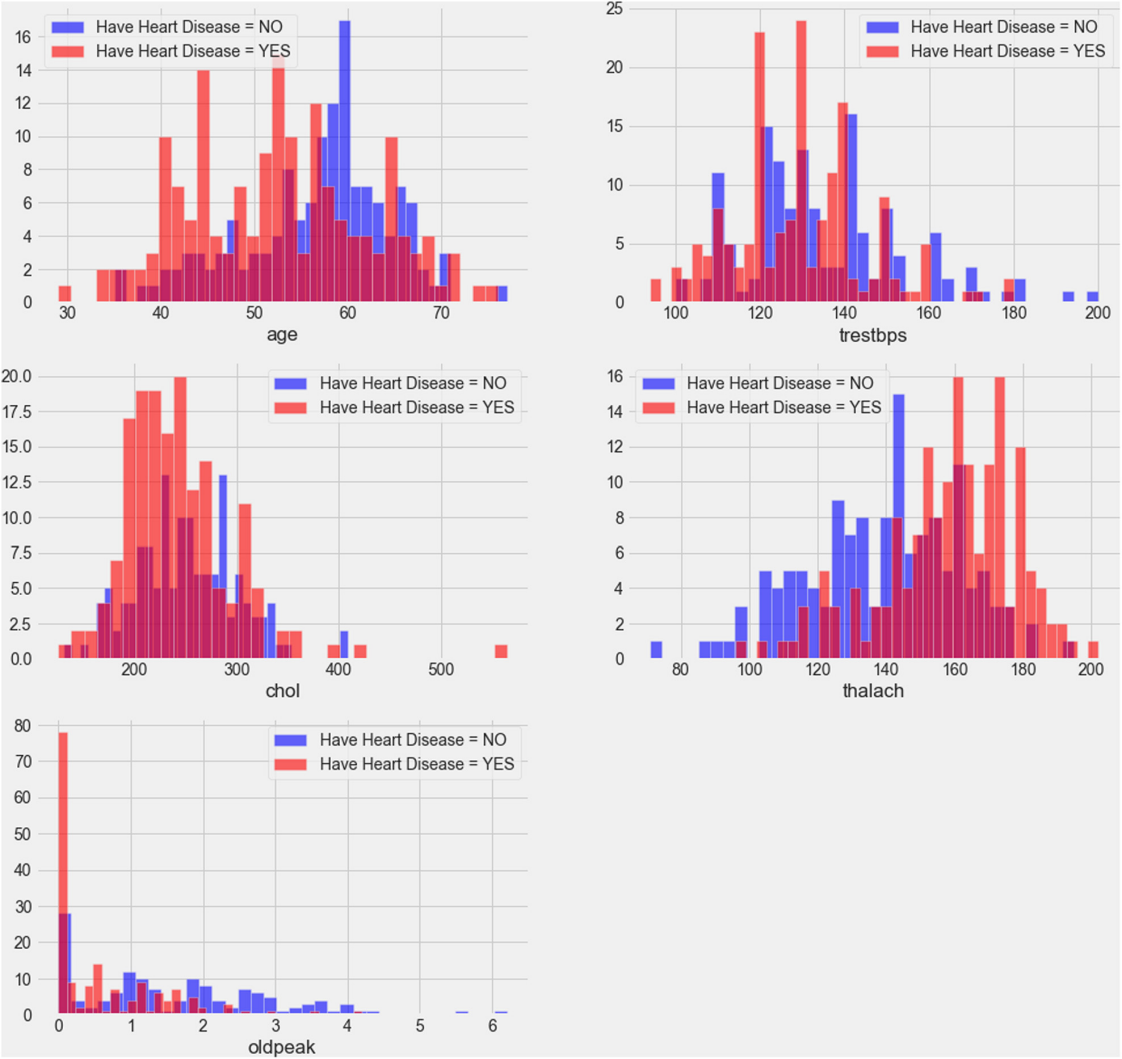
Correlation plot of CVD data set continuous features.

The data set contains both categorical and continuous features as explained in Figures 3 and 4. The data set consists of patients between the ages of 29 and 77. Pandas, NumPy, sklearn and matplotlib python libraries were used to analyse and visualize the data. Two standard and reliable MLP and *K*-NN ML methods were employed for binary classification (CVD or no CVD).

Figure 3 shows the relationship of categorical features with the target. The plot demonstrates that females have a higher probability of developing CVD than males, and people with cp values of 1, 2, or 3 have a greater probability of contracting CVD than people with a cp value of 0. People with fbs values greater than 120 have higher chances of having heart ailment than people with fbs values less than 120. People with a Restecg value of 1 have a higher probability of heart ailment than people with values of 0 and 2. Similarly, people with an exang value of 0 are more prone to CVD than people with a value of 1. People with a slope value of 2 have a higher probability of suffering from heart ailment than people with slope values of 0 and 1. People with a ca value of 0 have a greater chance of suffering from CVD than people with ca values of 1, 2, or 3, and people with a thal value of 2 have greater chances of contracting CVD than people with values of 1 and 3. The correlation plot in Figure 4 demonstrates the range of continuous feature values for which a person should be alerted to avoid CVD problems.

### 2.2 Pre-processing of CVD data

There are many missing and noisy data in public data sets. These data are pre-processed to minimize distorted measurements and make predictions more reliable. The pre-process stage involves smoothing, standardization, and aggregation. During the data pre-processing phase, the correlation matrix is used to identify the correlation between different CVD data set features and shows whether the features are positively or negatively associated with one another. After pre-processing of the data set, certain categorical variables such as cp, sex, chol, and trestbps are converted into dummy variables, and the resultant data are scaled before training the ML models. All variables were scaled down using standard normal distribution, and a cross-validation *K*-NN value of 20 was applied. For different *k* values between 1 and 21, different accuracy values were obtained.

#### 2.2.1 Scatter plot

The scatter plot (data visualization plot) is a mathematical diagram in cartesian coordinates showing the relationship between two variables of a given data set. It shows the relationship between two quantitative variables. If two variables lie on a line or curve, then they are correlated. Therefore, it objectively determines whether or not a particular cause and effect are related. In this study, various scatter plots are presented to identify potential root causes of CVD.

### 2.3 ML

ML is described as part of Artificial Intelligence (AI) in which a model acquires knowledge from past experience, without being explicitly programmed. Medical data are used in different ML classifiers for classification or forecasting of diseases. Supervised learning, which requires labelled input data for training the machine, is employed, and the machine learns with features called biomarkers of heart disease. Patient data are fed to the machine, and labelled outcomes are obtained. There are different ML classifiers for building data analysis models such as random forest, *K*-NN, and MLP. The main objective of each classifier is to build a model with exceptional disease detection capability. The classifiers used in this investigation, *K*-NN and MLP, have exceptional detection potentials.

#### 2.3.1 ML classifiers

##### 2.3.1.1 *K*-Nearest neighbour

*K*-NN is a non-parametric classifier used to determine whether a patient has CVD or not using a labelled known data set. Predictions are made based on *k* numbers of frequently used neighbours for a new object, and a different distance metric for finding the *K*-NN is used. *K*-NN classifies new training data points based on similarity measurements. Data points are classified by considering the majority of votes from its neighbours. This works effectively for small dimensional data sets. *K*-NN does not require extra training for classification if a new data point is added to the existing data set. It is an inefficient algorithm for large data sets and requires more memory space for computation and longer model testing times because of the need to compute the distance between training data set and testing data set during each test.

##### 2.3.1.2 Multi-layer perceptron (MLP)

**Figure 5:**
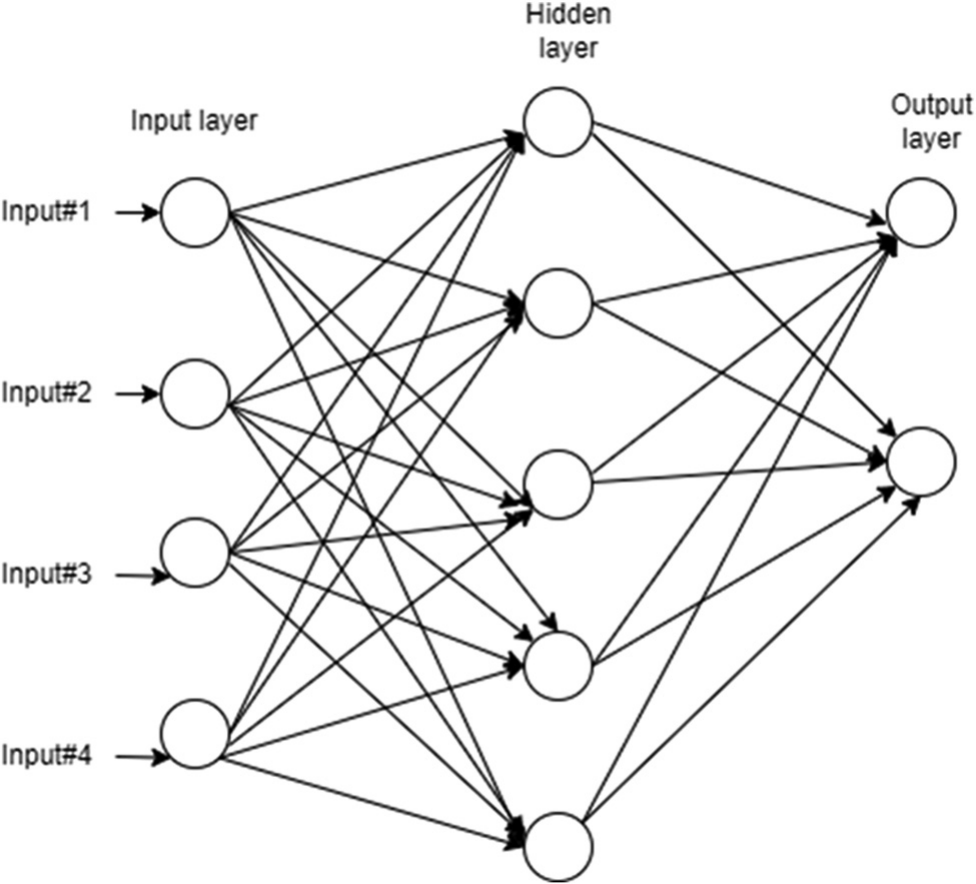
Schematic diagram of MLP.

This is a type of artificial neural network. It functions like a human brain, such that information passes from an input node to an output node via hidden nodes in a forward direction. It contains three nodes: input node, output node, and hidden node. It is one of the powerful supervised types of classifiers for the efficient prediction of chronic diseases. An MLP is the combination of a number of neural units known as perceptrons. In this model, each layer contains a number of weights via which perceptrons are connected with each other (Figure 5). To train the feed-forward neural network, a backpropagation learning algorithm is used. Weights are adjusted to minimize errors during neural network training [16].

Two types of activation functions are commonly used in the hidden layer: the sigmoid activation and tanh functions, given in equations (1) and (2), respectively. The weight updating rule for minimizing errors is given in equation (4).

(1) \text{Sigmoid function},\sigma \hspace{.25em}(x)=\frac{1}{1+{\text{e}}^{-x}},]

(2) \text{tanh}\hspace{.25em}(x)=2\sigma (x)(2x)-1,]

(3) \text{Mean square error}=\frac{1}{p}\mathop{\sum }\limits_{i=1}^{p}{({m}_{i}-\hat{m})}^{2},]

where *P* is the number of samples, *m* is the actual observation, \hat{m}] is the predicted sample value.

(4) \ast {w}_{j}={w}_{j}-\alpha \left(\frac{\text{d}E}{\text{d}{w}_{j}}\right),]

where *w_*j*_ is the updated weight, *w*_*j*_ is the previous weight*, α* is the learning rate (0 < *α* < 1) *E* is the error term (*m* – \hat{m}] ).

### 2.4 Performance evaluation of models

The potential of ML algorithms is assessed using a set of performance metrics. To evaluate the parameters, a confusion matrix including the true positive (TP), false positive (FP), true negative (TN), and false negative (FN) for actual and predicted data are obtained. A confusion matrix is a table frequently used to determine performances of classifiers. Table 1 explains these parameters.

**Table 1:** Definition of confusion matrix parameters

Confusion matrix parameters	Description
True positive	Instances where we predicted yes (patient has the CVD), and it turned out to be correct
True negative	Instances where a patient does not have CVD and was predicted to not have CVD
False positive	Instances where a patient does not have CVD, but was predicted to have CVD
False negative	Instances where a patient does not have CVD and was predicted to not have CVD

The performance of each classifier is measured using the confusion matrix and determined by five parameters: classification accuracy, sensitivity, specificity, *F*1-score, support, and receiver operating characteristics (ROC) – area under the curve (Table 2). The classification accuracy is used to determine the percentage of cases correctly classified and is calculated using four parameters: TP, TN, FP, and FN. The sensitivity signifies what percentage of patients with CVD is correctly identified. The specificity indicates the percentage of patients without CVD and those correctly classified. The *F*1-score takes the harmonic mean of a classifier’s precision and recall to create a single statistic, and is mostly used to compare the performance of two different classifiers.

**Table 2:** Definition of performance metrics

Performance metrices	Definition/explanation
Accuracy	\frac{\text{True}\hspace{.25em}\text{positive}(\text{TP})+\text{True}\hspace{.25em}\text{negative}(\text{TN})}{\text{True}\hspace{.25em}\text{positive}(\text{TP})+\text{True}\hspace{.25em}\text{negative}(\text{TN})+\text{False}\hspace{.25em}\text{positive}(\text{FP})+\text{False}\hspace{.25em}\text{negative}(\text{FN})}]
Precision	\frac{\text{TP}}{\text{TP}+\text{FP}}]
Recall (TP rate)	\frac{\text{TP}}{\text{TP}+\text{FN}}]
*F*1-score	\frac{\text{TP}\cdot \text{TP}}{\text{TP}+\text{TP}+\text{FP}+\text{FN}}]
Support	The number of actual occurrences of a class in the provided data set
FP rate	\frac{\text{FP}}{\text{FP}+\text{TN}}]
Area under the curve (AUC)	AUC is an important feature of the ROC curve that measures the ability of a classifier to distinguish between classes. The greater the AUC, the better the model’s performance
ROC	An ROC, or ROC curve, is a graphical representation of a binary classifier
Macro average	All classes equally contribute to the final averaged metric
Weighted avg.	The weight of each class’s contribution to the average

## 3 Results

**Figure 6:**
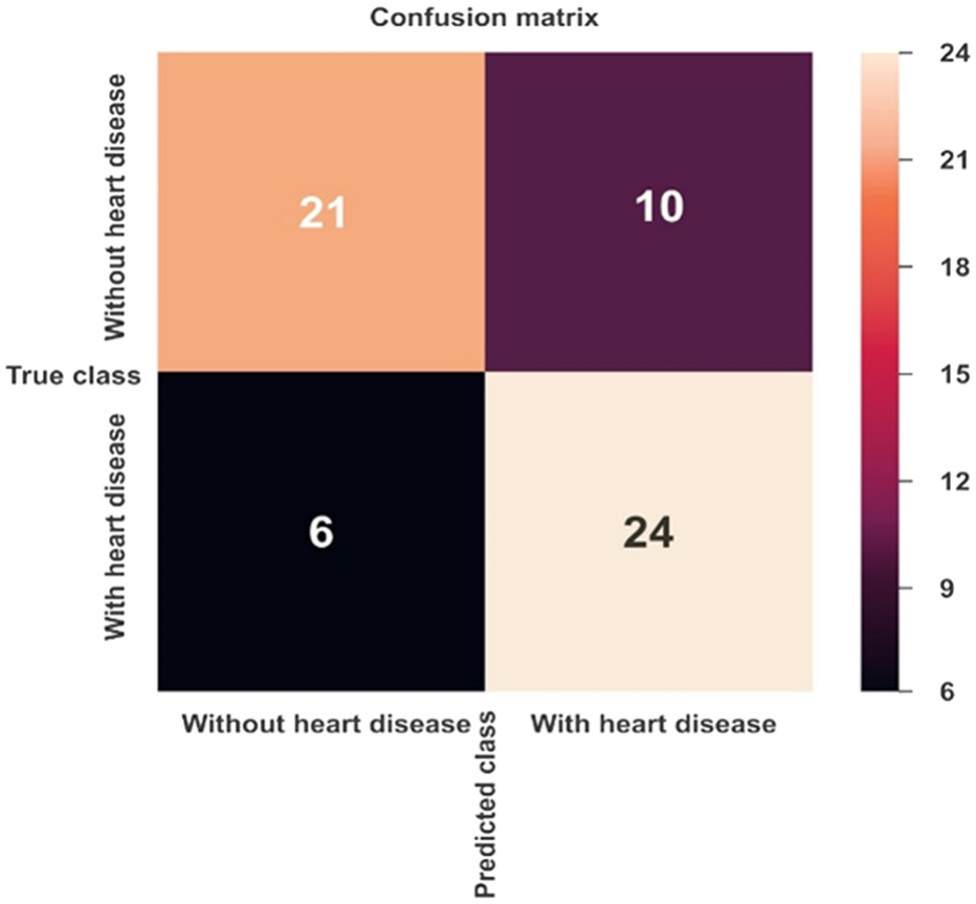
Confusion matrix for CVD prediction using the *K*-NN model.

**Figure 7:**
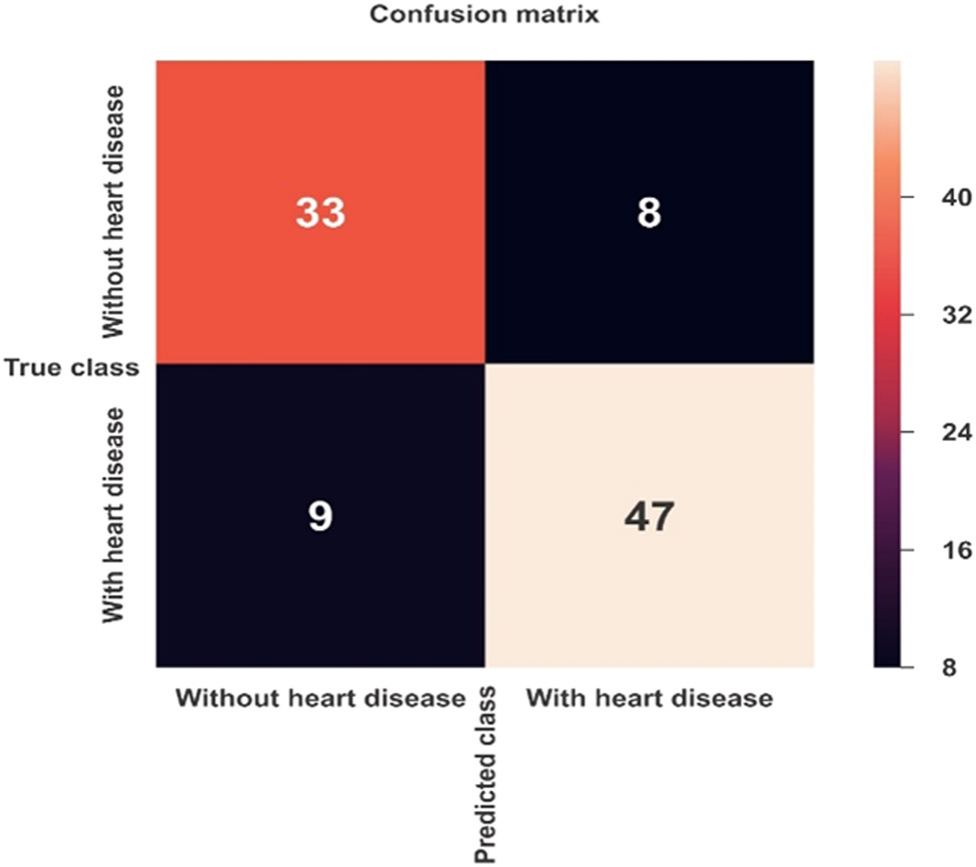
Confusion matrix for CVD prediction using the MLP model.

Figures 6 and 7 show the confusion matrix of *K*-NN and MLP ML models, respectively. Table 3 shows the combined confusion matrix, which reveals that between the two approaches, the MLP model predicted more TPs (47 vs 24), more TNs (33 vs 21), more FPs (9 vs 6), and fewer FPs (8 vs10).

**Table 3:** Confusion matrix results of ML models

Confusion matrix parameters	ML algorithms	
	*K*-NN	MLP
TN	21	33
TP	24	47
FP	10	8
FN	6	9

### 3.1 Scatter plot between features of CVD

**Figure 8:**
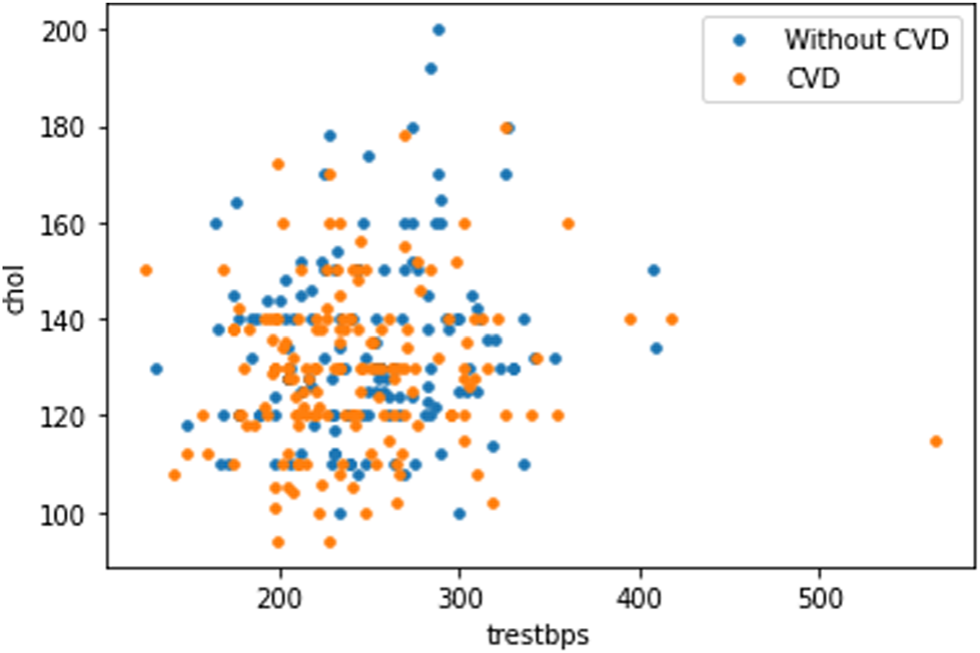
Data visualization plot showing correlation between features.

**Figure 9:**
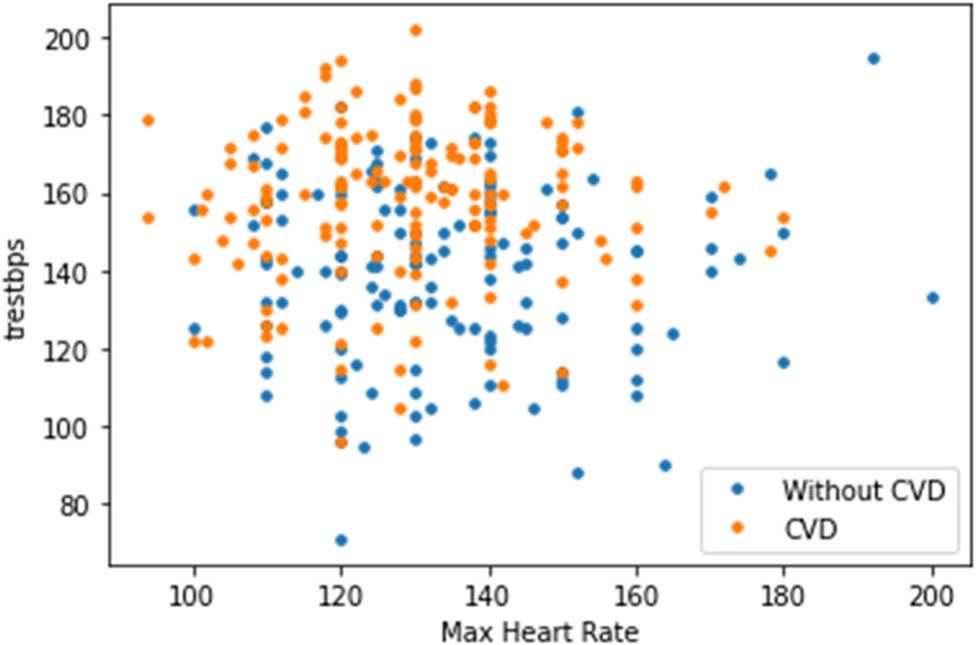
Data visualization plot showing a correlation between resting blood pressure and maximum heart rate.

**Figure 10:**
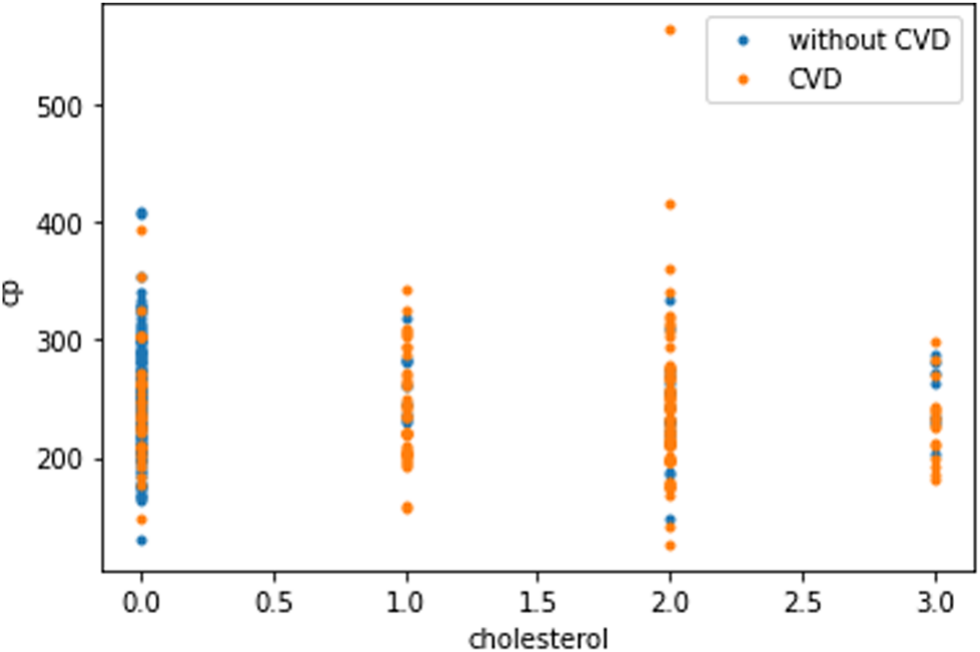
Data visualization plot showing a correlation between chest pain and cholesterol level.

**Figure 11:**
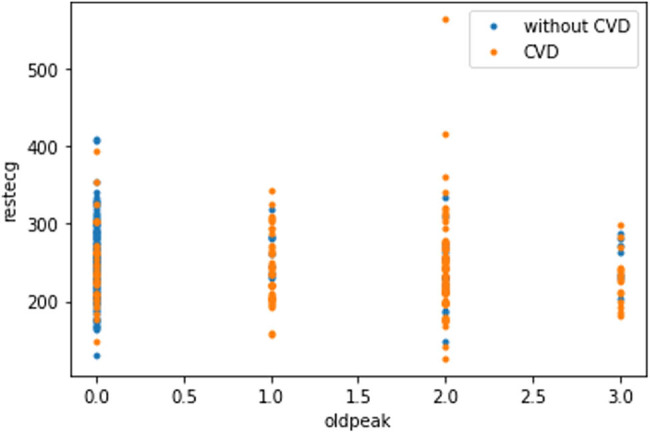
Data visualization plot showing a correlation between resting electrocardiographic and old peak.

**Figure 12:**
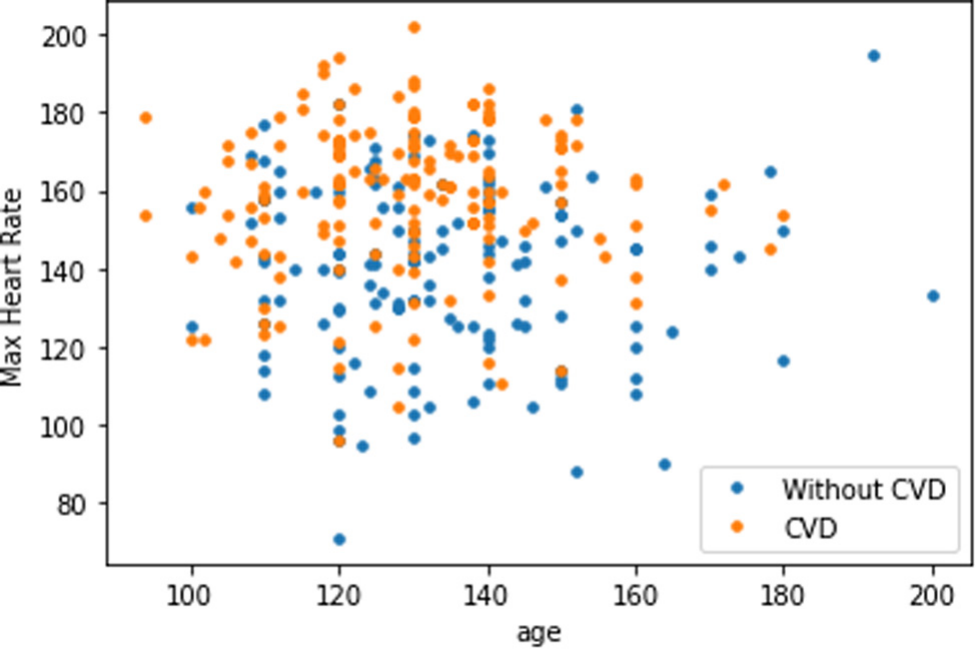
Scatter plot of maximum heart rate and age.

**Figure 13:**
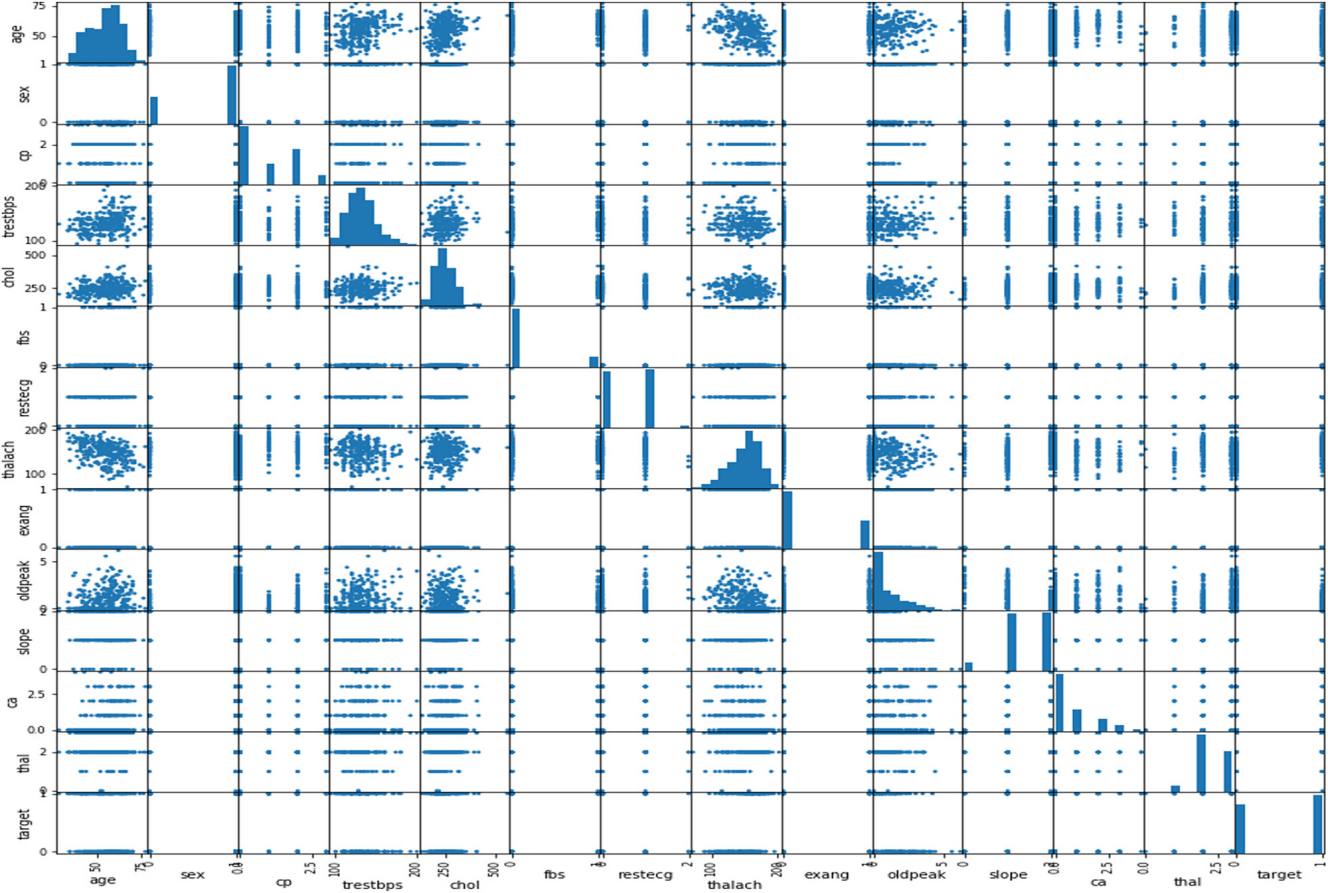
Scatter plot showing correlation of CVD data set features.

The plot in Figure 8 shows that as the cholesterol (chol) level of a person increases, blood pressure (trestbps) rises, which consequently increases the probability of one suffering from CVD. The plot in Figure 9 indicates that with the rise of blood pressure (trestbps), the heart rate (max heartbeat) of a person increases, which is a sign of heart ailment. Figure 10 shows that there is a strong correlation between chol and cp levels. The relationship between restecg and old peak is shown in Figure 11. In Figure 8, the orange colour signifies the people suffering from CVD while the blue colour represents people not suffering from CVD. The scatter plot in Figure 12 shows that people with heart rates above 140 between the ages of 40 and 55 are more prone to CVDs. A data visualization plot showing the correlation between individual features is shown in Figure 13.

### 3.2 Correlation plot between variables of the data set

**Figure 14:**
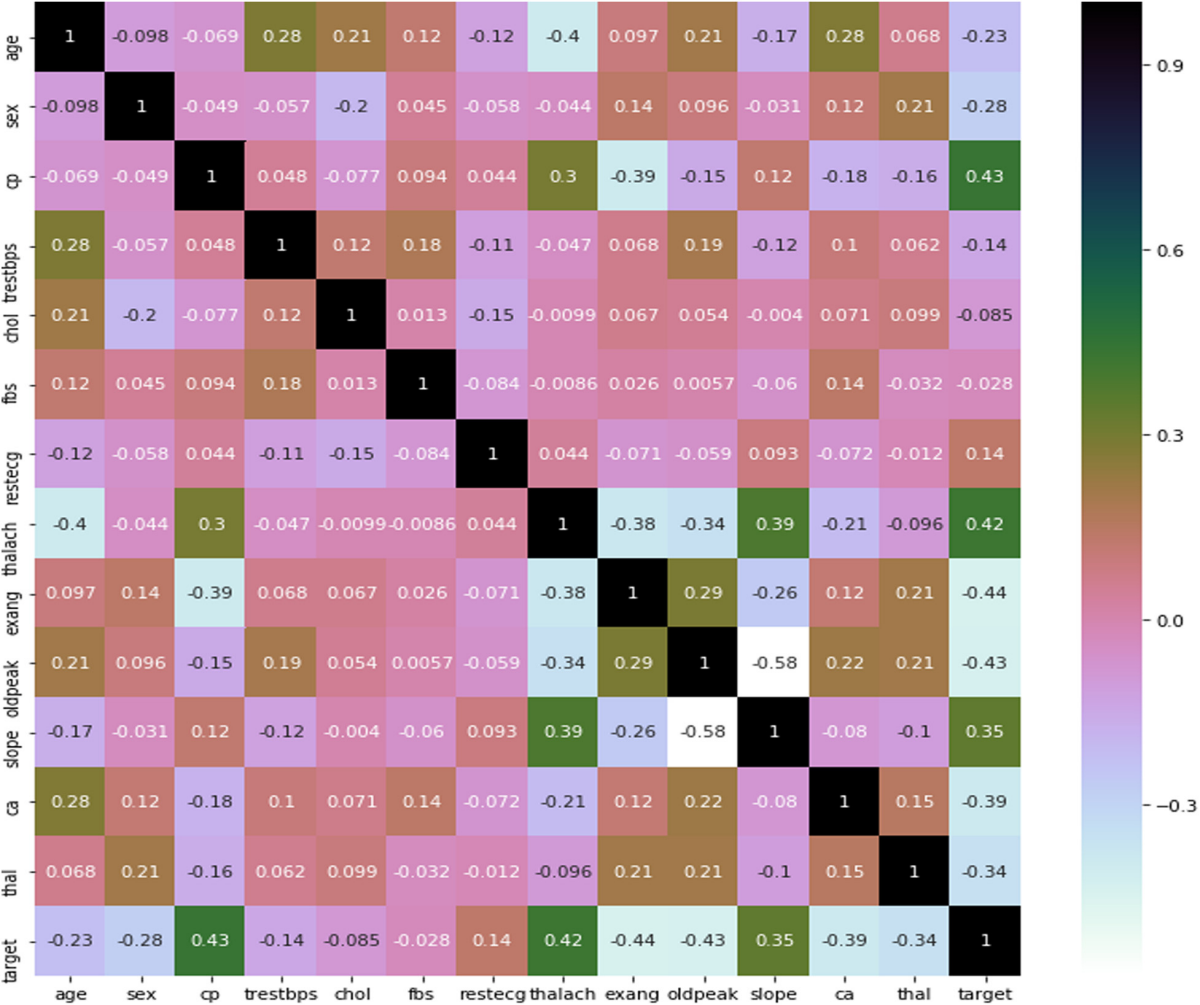
Correlation matrix plot between features.

The correlation matrix plot (Figure 14) shows that cp has a positive correlation with goal output; with a value of 0.43, it indicates that if a person has cp, he is likely to have CVD. Similarly, some traits are negatively connected, such as cholesterol and the target variable; their correlation value of –0.085 indicates that a person with cholesterol has suffered from CVD. It is always preferable to choose features that have positive correlations with the target variable. Features with values close to 0.9 or greater than 0.6 are substantially correlated with each other, as are features with values close to –0.3. These factors can be eliminated during data collection. For example, thalach and oldpeak have a significant relationship with a correlation value of –0.34. During data pre-processing, one of these features can be removed.

### 3.3 Performance evaluation of ML models

**Figure 15:**
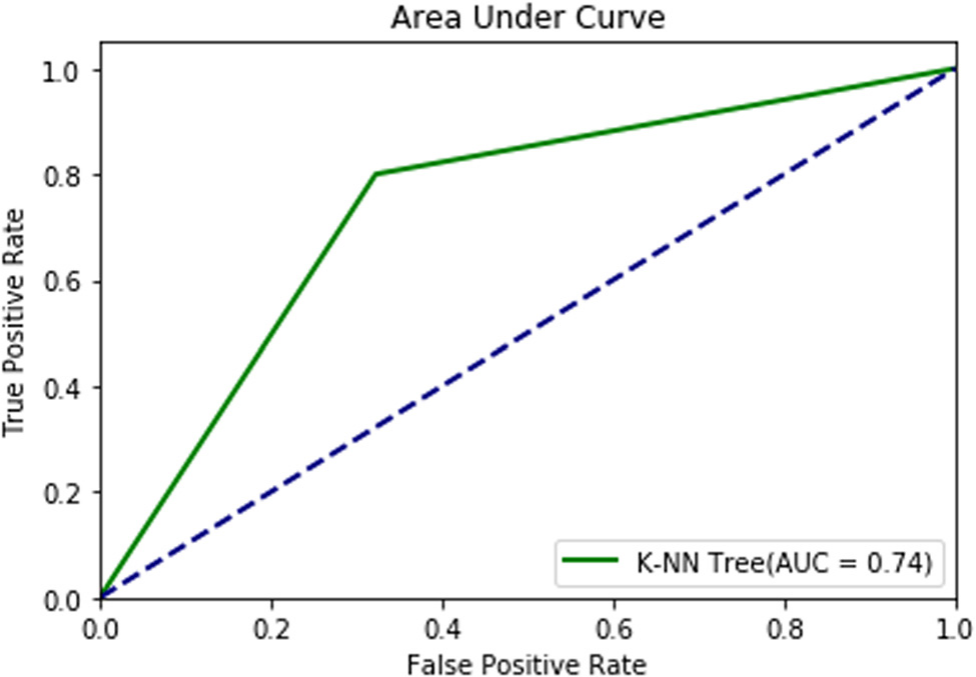
ROC plot of the *K*-NN model.

Figure 15 shows the ROC plot of the *K*-NN model with an AUC score of 74%, which signifies that the model can identify 74% of CVD cases accurately.

**Figure 16:**
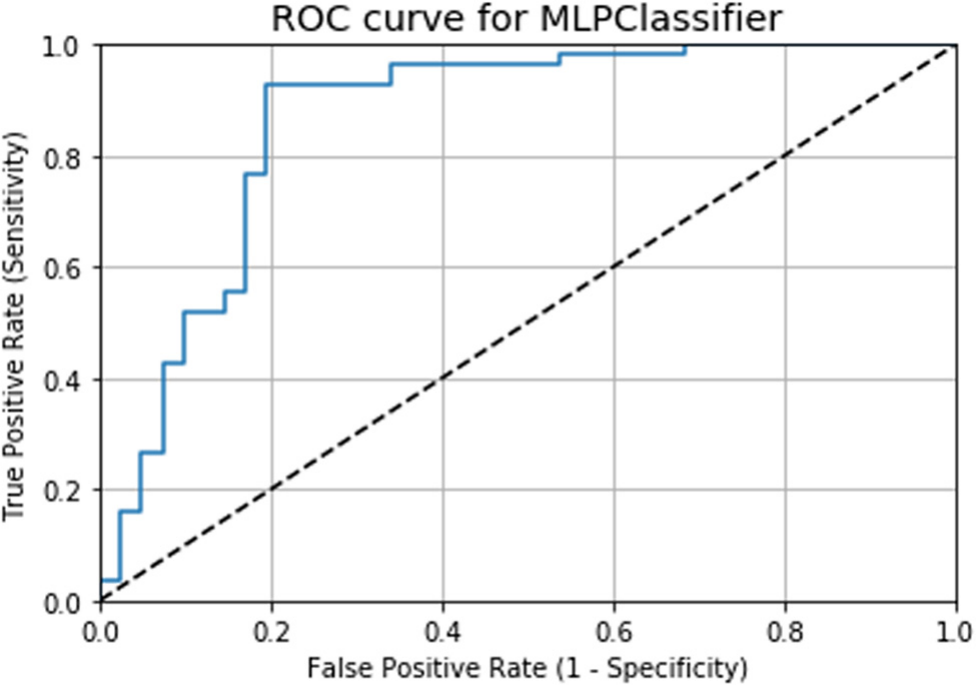
ROC curve for the MLP model.

From the classification results of the *K*-NN model given in Table 4, performance parameters such as precision, recall, *F*1-score, and support values for a target output of 0 are 0.78, 0.68, 0.72, and 31, respectively. Similarly, for a target output of 1, the precision, recall, *F*1-score, and support values are 0.71, 0.80, 0.75, and 30, respectively. The ROC in Figure 16 shows the diagnostic capability of the MLP model, which has a diagnostic accuracy of 86.41% in CVD prediction. From the classification results, the precision, recall, *F*1-score, and support values for a target output of 0 are 0.79, 0.80, 0.80, and 41, respectively. However, for a target output of 1 using the MLP model, the precision, recall, *F*1-score, and support values are 0.85, 0.84, 0.85, and 56, respectively (Table 5).

**Table 4:** Classification results of the *K*-NN model

Parameter	Precision	Recall	*F*1-score	Support
0 (Without CVD)	0.78	0.68	0.72	31
1 (With CVD)	0.71	0.80	0.75	30
Accuracy	NA	NA	0.74	61
Macro average	0.74	0.74	0.74	61
Weighted average	O.74	0.74	0.74	61

**Table 5:** **C**lassification results of the MLP model

Parameter	Precision	Recall	*F*1-score	Support
0 (Without CVD)	0.79	0.80	0.80	41
1 (With CVD)	0.85	0.84	0.85	56
Accuracy	NA	NA	0.82	97
Macro avg	0.82	0.82	0.82	97
Weighted avg	0.83	0.82	0.83	97

### 3.4 Comparison of results for both the models

The accuracy and AUC scores of the *K*-NN and MLP algorithms are presented in Table 6. The obtained accuracy (82.47%) and AUC (86.41%) values for the MLP model are higher compared to those of the *K*-NN model. This shows that the MLP model predicts CVD more accurately. Similarly, the AUC score for the diagnosis rate of the *K*-NN model is 86.21%, while that of the MLP model is 86.41%.

**Table 6:** Comparision of accuracy and AUC scores obtained from ML models

ML algorithms	Accuracy score (%)	AUC score (%)
*K*-NN	73.77	86.21
MLP	82.47	86.41

The comparison of results indicates that the MLP model has a higher prediction accuracy of 82.47%, followed by the *K*-NN model with an accuracy value of 73.77%. The accuracy comparison of these two models is presented in Fig. S1. The ROC plot also shows that the MLP algorithm has a higher AUC value (86.41%) compared to the *K*-NN model (86.21%). The AUC comparison for the *K*-NN and MLP models is shown in Fig. S2.

## 4 Discussion

A comparison of accuracy values from existing studies is shown in Table 7. The simulation result of the proposed work using the MLP algorithm is 82.47%, compared to 47.54% obtained by Kaur et al. [17]. The comparison of results demonstrates that the MLP algorithm provides a higher accuracy (82.47%) when compared to that of Naïve Bayes (69.11%) [18] and Decision tree (78.57%, 80.68%) [19,20]. Therefore, the proposed MLP model is more efficient in CVD prediction when compared to other ML algorithms. By increasing the number of hidden layer nodes and employing a 10-fold cross-validation technique in the MLP model, we are able to improve its accuracy.

**Table 7:** Performance comparision of the proposed work with existing works

Existing work	Algorithms	Accuracy (%)	Reference
Kaur et al., 2019	MLP	47.54	[17]
Nahar et al., 2013	Naïve Bayes	69.11	[18]
Verma et al., 2016	Decision Tree	80.68	[19]
Ei-bialy et al., 2015	Decision Tree	78.54	[20]
Proposed work	MLP	82.47	—

Regarding the literature, Alizadehsani [21] compared the performances of various well-known ML techniques for coronary artery disease detection. Latha and Jeeva [22] proposed a model for increasing the accuracy of weak classifiers by 7.26% using an ensemble model. Ahmed and co. [23] reported a heart disease risk-prediction model with a 94.9% accuracy using random forest. Beunza et al. [24] compared the performance of several ML algorithms based on the Framingham heart database to predict coronary heart disease using R-Studio and Rapid Miner and achieved the highest AUC value of 0.75 using a support vector machine method. Kim et al. [25] obtained a 0.89 AUC value using an artificial neural network to predict the survival rate of injured patients. In another study, Shah et al. [26] obtained a maximum accuracy of 90.78% when predicting heart disease using the *K*-NN algorithm, and Pal and Parija [27] reported a heart disease risk-prediction model with 86.9% accuracy, 90.6% sensitivity, and 82.7% specificity by using the random forest algorithm.

This study presents a comparison of two ML techniques for CVD prediction: *K*-NN and MLP. Between these algorithms, MLP provides better accuracy (82.47%) than *K*-NN with an accuracy of 73.77%. The diagnosis rate was found to be 86.41 and 86.21% for the MLP and *K*-NN algorithms, respectively. In the medical field, the diagnosis procedure for CVD is costly and time-consuming. The proposed approach suggests that ML can be used as a clinical tool in the detection of CVD and will be particularly useful for physicians in the event of a misdiagnosis. The constructed MLP model offers consistent accuracy compared to other techniques mentioned and is also capable of predicting other diseases. In this study, the performance of the model was improved by removing attributes with null values using an explorative data analysis method and by increasing the number of hidden layer nodes. The proposed method is expected to assist in the further development of the healthcare field. The proposed method can also be used for the classification of other chronic diseases such as breast cancer, liver disease, diabetes mellitus, and thyroid. The developed models can be applied to large data sets to predict chronic diseases using IoT and cloud computing techniques. From the above analysis, the application of ML techniques will vastly aid in preventing fatalities and supplement the efforts of doctors in fighting CVD occurrence among all patient categories of different age groups, genders, and socio-economic backgrounds. If implemented, this would be a classic case of new-age technology application for the benefit of mankind.

## Figures and Tables

**Figure A1:**
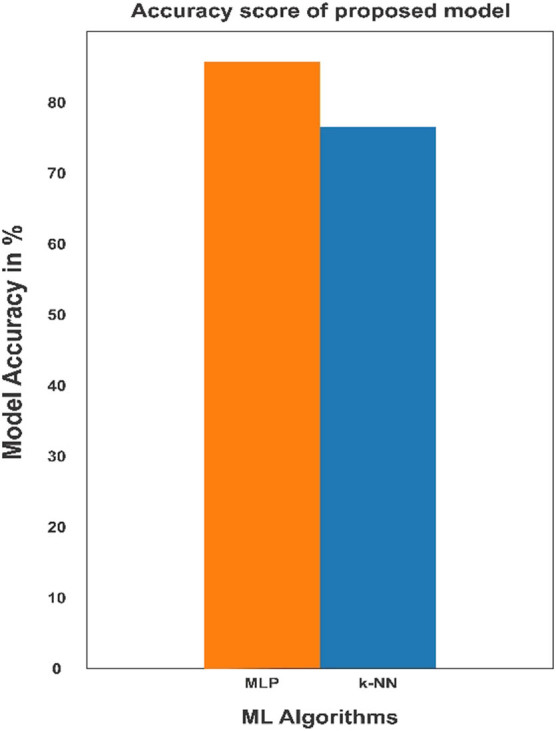
Comparision of accuracy score of ML models.

**Figure A2:**
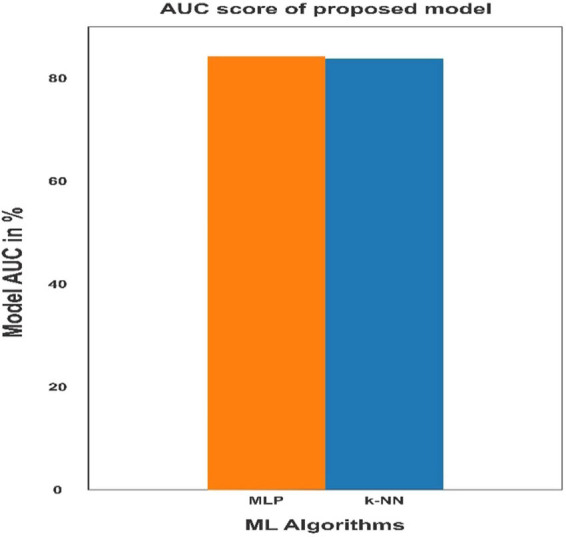
Comparison of AUC score of ML models.

## Appendix

**Table A1:** Description of features of the dataset

SL NO.	Features	Types	Description	Range of features increasing the probability of heart disease
1.	Age	Continous	Age in years	NA
2.	Sex	Categorical	Male,Female	1=male,0=female
3.	Cp	Categorical	Chest pain type0: Typical angina1: Atypical angina2: Non-anginal pain3.Asymptomatic	People with cp equl to 1, 2, 3 are more likely to have heart disease than people with cp equal to 0.
4.	Trestbps	Continous	Resting blood pressure in mm Hg	Concerning above 130-140 .
5.	Chol	Continous	Serum cholesterol in mg/dl	serum = LDL + HDL + .2 * triglyceridesAbove 200 is matter of concern
6.	Fbs	Categorical	Fasting blood sugar>120 mg/dl	1=true,0=false>126 mg/dl signals diabetesPeople with Fbs equal to 1 increased the probability of suffering from heart disease than people with Fbs equal to 0.
7.	Restecg	Categorical	Resting electrocardiographic results. People with value 1 (signals non-normal heart beat, can range from mild symptoms to severe problems) are more likely to have heart disease.	0: Nothing to note1: Non normal heart beat can range from mild symptoms to severe problems2: Possible or definite left ventricular hypertrophyEnlarged heart's main pumping chamber
8.	Thalach	Continous	Maximum heart rate achieved	More than 140 are more likely to have heart disease.
9.	Exang	Categorical	Exercise induced angina	People with value 0have more probability of suffering from heart disease than people with value 1 1=yes0=no
10.	Oldpeak	Continous	ST depression induced by exercise relative to rest looks at stress of heart	During exercise unhealthy heart will stress more
11.	Slope	Categorical	The slope of the peak exercise ST segment	0: Upsloping: better heart rate with excercise(uncommon)1: Flatsloping: minimal change (typical healthy heart)2: Downsloping: signs of unhealthy heart
12.	Ca	Categorical	Number of major vessels (0-3) colored by flourosopy	Ca equal to 0 are more likely to have heart disease.
13.	Thal	Categorical	Thalium stress result	1,3: normal6: fixed defect: used to be defect 7: reversable defect: no proper blood movement when exercising People with thal value equal to 2 more likely to have heart disease.
14.	Target	Categorical	Have heart disease or not	1=yes, 0=no
